# A multicenter study investigating the molecular fingerprint of psychological resilience in breast cancer patients: study protocol of the SCAN-B resilience study

**DOI:** 10.1186/s12885-018-4669-y

**Published:** 2018-08-06

**Authors:** Ulrika Axelsson, Lisa Rydén, Per Johnsson, Patrik Edén, Johanna Månsson, Ingalill Rahm Hallberg, Carl A. K. Borrebaeck

**Affiliations:** 1 Department of Immunotechnology and CREATE Health Translational Cancer Center, Lund University, Medicon Village (Bldg 406), 223 81 Lund, Sweden; 2 Department of Clinical Sciences Lund University, Surgery and Department of Surgery Skåne University Hospital, Lund, Södra Förstadsgatan 1, 214 28 Malmö, Sweden; 3 Department of Psychology, Lund University, Box 213 221 00, LUND, Sweden; 4 Computational Biology and Biological Physics, Department of Astronomy and Theoretical Physics, Lund University, 223 62 Lund, Sweden; 5 Department of Health Sciences, Lund University, Lund, Sweden; 6 Pufendorf Institute, Lund University, 221 00 Lund, SE Sweden

**Keywords:** Psychological resilience, Body and mind, Biomolecular parameters, Breast cancer, Quality of life, CD-RISC, SF36, Epigenetics

## Abstract

**Background:**

Individual patients differ in their psychological response when receiving a cancer diagnosis, in this case breast cancer. Given the same disease burden, some patients master the situation well, while others experience a great deal of stress, depression and lowered quality of life. Patients with high psychological resilience are likely to experience fewer stress reactions and better adapt to and manage the life threat and the demanding treatment that follows the diagnosis. If this phenomenon of mastering difficult situations is reflected also in biomolecular processes is not much studied, nor has its capacity for impacting the cancer prognosis been addressed.

This project specifically aims, for the first time, to investigate how a breast cancer patient’s psychological resilience is coupled to biomolecular parameters using advanced “omics” and, as a secondary aim, whether it relates to prognosis and quality of life one year after diagnosis.

**Method:**

The study population consists of newly diagnosed breast cancer patients enrolled in the Sweden Cancerome Analysis Network – Breast (SCAN-B) at four hospitals in Sweden. At the time of cancer diagnosis, the patient fills out the standardized method to measure psychological resilience, the “Connor-Davidson Resilience scale” (CD-RISC), the quality of life measure SF-36, as well as providing social and socioeconomic variables. In addition, one blood sample is collected. At the one-year follow-up, the patient will be subjected to the same assessments, and we also collect information regarding smoking, exercise habits, and BMI, as well as patients’ trust in the treatment and their satisfaction with the care and treatment.

**Discussion:**

This explorative hypothesis-generating project will pave the way for larger validation studies, potentially leading to a standardized method of measuring psychological resilience as an important parameter in cancer care. Revealing the body-mind interaction, in terms of psychological resilience and quality of life, will herald the development of truly personalized psychosocial care and cancer intervention treatment strategies.

**Trial registration:**

This is a retrospectively registered trial at ClinicalTrials.gov, ID: NCT03430492 on February 6, 2018.

**Electronic supplementary material:**

The online version of this article (10.1186/s12885-018-4669-y) contains supplementary material, which is available to authorized users.

## Background

Cancer is a traumatic experience that completely interrupts the mental balance in the life of an individual. However, some individuals diagnosed with cancer, of any kind, are seemingly more successful in processing and adapting to this life threat than others [1]. This improved outcome cannot only be explained by the severity of the cancer and the treatment. These patients also score better on standardized measures capturing psychological resilience [2].

It has been reported that a low psychological resilience increases the risk of feeling hopeless [2] and that hopelessness, powerlessness, and meaninglessness have an impact on the function of the brain, indicating a body-mind interaction [3]. Furthermore, a variety of stressors, such as trauma, depression, and social isolation, have been shown to be associated with the dysregulation of various neuroendocrine hormones such as catecholamines and cortisol [4]. Elevated levels of norepinephrine, due to stress, have been shown to increase the level of matrix metalloproteinease-9 (MMP-9). Clinically, both depression and stress have been related to MMP-9 secretion by tumor-associated macrophages (TAM) in patients with ovarian cancer. Since TAM promote a proinflammatory tumor environment, the effect of stress on TAM have significant implications for tumor progression [5]. Consequently, the reaction to a stressor such as cancer is a physical reaction as well as a mental experience, and it is evident that psychosocial and behavioral factors affect cancer progression [6]. A recent review [7] describes and clarifies that biobehavioral factors not only affect cellular immunity but both directly and indirectly modulate fundamental processes in cancer growth, including inflammation, angiogenesis, invasion, and metastasis. Consequently, it is reasonable to conclude that knowledge of the patient’s resilience is a prerequisite for the capability of performing individualized cancer care. However, little is known regarding how to identify the most vulnerable patients as well as the relation to biomolecular processes. This clearly shows why it is of utmost importance to elucidate the body and mind interactions in cancer patients.

Consequently, this project will apply a bio-psychosocial approach to explore the body and mind interaction in patients diagnosed with breast cancer. Our hypothesis is that patients displaying a high psychological resilience, according to a standardized well-accepted method, the “Connor-Davidson Resilience Scale” (CD-RISC) [8, 9], i.e., low stress reactions, low hopelessness and low fatigue, also present a specific pattern of bimolecular signatures. In addition, our hypotheses are that the grade of resilience also influences the quality of life, which eventually can be translated into a prognosis for the individual patient. The aim is to decipher the biology that correlates with the grade of psychological resilience and to investigate the psychological resilience longitudinally (initially, the first year from diagnosis) and relate it to disease burden, quality of life (QoL) and survival. Such knowledge will enable us to start developing and applying evidence-based interventions to those at high risk of stress-related complications during disease progression.

### Psychological resilience

Psychological resilience is a concept bringing together the bio-psychosocial resistance that helps a person to address a trauma such as cancer. It has been defined as *“a dynamic process in which individuals adjust and cope in an adaptive manner when confronted with significant and threatening adversity”* [10]. Thus, psychological resilience, from a bio-psychosocial perspective, is a protective factor. There are other similar concepts, such as sense of coherence [11]; however, these methods are not validated to the same extent as the CD-RISC [8, 9].

## Methods and design

### Purpose

The purpose of this study is to define the association between psychological resilience and biomolecular signatures in cancer patients and to relate psychological resilience to prognosis as well as quality of life, as this could potentially reveal a novel avenue of therapeutic interventions, medical as well as psychosocial, from the perspective of “you can only treat what you can measure.”

### Scientific approach

This project applies a multifactorial approach that combines bio-psychosocial assessment and clinical data with advanced genomics and proteomics technologies. The primary endpoint for this study is to define the association between psychological resilience and biomolecular signatures in breast cancer patients. A second aim is to investigate whether psychological resilience relates to QoL one year after diagnosis, and a third overall long-term goal of this study is to determine if psychological resilience relates to prognosis, as this could potentially open up a novel avenue of therapeutic interventions in cancer, medical as well as psychosocial.

### Study population

The present study, denoted “*SCAN-B Resilience*” (**Ethical approval 2009/658**), as a part of the Sweden Cancerome Analysis Network – Breast (SCAN-B) initiative [12], is a prospective breast cancer study with an established infrastructure for enrollment and follow-ups for patients. In addition to the main ethical approval, the following amendments are also approved: **2010/383** (expansion of sites for SCAN-B, including Blekinge County Hospital, Central Hospital Växjö and Hallands Hospital Halmstad), **2012/58** (updated patient information for SCAN-B v3), **2015/277** (updated patient information for SCAN-B v4), **2015/522** (The SCAN-B Resilience study), **2017/875** (The SCAN-B Resilience study in Helsingborg) and **2017/88 (**The one-year follow up of SCAN-B Resilience). The SCAN-B Resilience, as part of SCAN-B, consequently addresses a well-defined cohort of women with breast cancer, facilitating the project and interpretation of the psychological resilience parameter. The study population is newly diagnosed breast cancer patients enrolled in SCAN-B at the Blekinge County Hospital, Central Hospital Växjö, Hallands Hospital Halmstad and Helsingborg Hospital. Karlskrona, Växjö and Halmstad are all urban cities with rural areas included in the patient uptake; thus, they are quite similar habitats. Helsingborg is a more densely populated city. Additional questions are added in the clinical research form (CRF) to enable consideration of the patient’s socioeconomic situation.

At all four hospitals, the study design works well. The personnel have a good routine of how to ask the patient to consent to the study and to give them time to fulfill the questionnaires. The blood sampling is performed together with the routine sampling for SCAN-B, resulting in no extra efforts for the patient or the personnel. In case of work overload, the personnel are free not to include a patient, which has occurred on a few occasions, mainly during summer vacations.

To investigate how the study population relates to all women with breast cancer, a comparison with the INCA (Information Network for CAncer care) register was performed. INCA is the National Swedish Quality Register on Breast Cancer. The INCA register includes approximately 100% of all women diagnosed with breast cancer in Sweden. Based on a comparison with cancer registrations in INCA for 2011–2016, 87% of all new breast cancer diagnosed women are included in SCAN-B [12, 13]. In February 2018, 70% of patients included in SCAN-B at Blekinge County Hospital, Central Hospital Växjö, and Hallands Hospital Halmstad were also enrolled in SCAN-B Resilience. Consequently, a majority (61%) of women diagnosed with breast cancer are enrolled in this study. Despite this level of participation, one might speculate about the 40% not included; some of these are excluded due to language problems (this applies to the SCAN-B study as well) or because of other circumstantial events such as shock. Possibly, some women’s unwillingness to participate is correlated to their psychological resilience, the rationale being less energy to participate due to low resilience, and thus the patient material in this study could be biased towards high-resilience patients.

### Data sources

The data collected in SCAN-B Resilience includes all data specifically collected for the study but also data collected in SCAN-B. In total, the data sources in the study is summarized in Table 1.

**Table 1:** Summary of data sources

**Data specific for SCAN-B Resilience**		
CD-RISC [8, 9]	At diagnosis	At one-year follow up
SF-36 [13 14]	At diagnosis	At one-year follow up
CRF diagnosis	At diagnosis	At one-year follow up
CRF follow-up	–	At one-year follow up
Clinical serology data	At diagnosis • Inflammation markers (e.g., IL8, IL6, CRP) • Stress hormones, such as cortisol, etc.	–
**Data available from SCAN-B**		
Patient Chart		
	To complement missing data from the registry	
**Data extraction of parameters available in INCA**		
Menstrual status		
Diagnostics	Localization	
	Date of visit at clinic	
	Date for suspicion of disease	
	Date of diagnosis	
	Screening discovery (Y/N)	
	Determined malignancy before surgery	
	Mammographic report	Date/Grade/Size
TNM classification		
Neoadjuvant treatment		
Surgery	Date	
	End result and complications	
	Type of breast surgery	Mastectomy/Partial mastectomy
Morphology		
Tumor biology		
Postoperative evaluation		
Postoperative treatment	Systemic treatment	
	Radiotherapy	
	Targeted therapy	
**Data from the National Board of Health and Welfare**		
Antibiotics		
Hormone treatment		
Inpatient care		
Outpatient care		
Cause of death register		

### Time plan

**2016–2018:** Enrollment of patients: At the time of cancer diagnosis, the patient fills out the standardized method to measure psychological resilience, the CD-RISC [8, 9], the quality of life measure Short Form (36-item) Health Survey (SF-36) [14, 15], as well as social and socioeconomic variables. These questions are shown in detail in an additional file (see Additional file 1). In addition, a blood sample is collected and specifically designated for SCAN-B Resilience.

**2017–2019:** Collection of data at one-year follow-up: The patient will be subjected to the same assessments as discussed above except for an additional blood sample. In addition, three questions capturing the patients’ trust in the treatment and satisfaction with the care and treatment are collected, and three questions regarding smoking and exercising, as well as weight and length are included. These questions are shown in detail in an additional file (see Additional file 2).

### Methods

#### Bio-psychosocial assessment

**Connor-Davidson Resilience Scale (CD-RISC)** is a standardized instrument most commonly used to measure psychological resilience, and to our knowledge, it is frequently used in studies where psychological resilience is measured in relation to health problems of various kinds, including cancer [16]. The instrument’s reliability is further evidenced by the fact that during 2003–2014, CD-RISC was used in over 300 publications, showing it to be a reliable and psychometrically sound instrument [16–18]. CD-RISC is composed of 25 items that altogether capture five factors assumed to form the person’s psychological resilience. The response format is on a Likert scale from disagree to agree, ranging from 1 to 5. The permission to use the CD-RISC has been obtained from Dr. Davidson.

**Short Form (36-item) Health Survey (SF-36)** [14, 15] is a well-known quality of life measure that has been used in many populations, thus providing data for comparison. The items are grouped into eight multi-item health concepts, where the response format is a yes or no alternative and a three- to six-response scale. Each of the health concepts are coded, summed and transformed to a 0 to 100 scale. The SF-36 has been translated and tested extensively in Sweden, and thus comparative data are available [19]. The permission to use SF-36 has been obtained from Quality Metric Incorporated.

**Clinical research form (CRF)** In addition to the above assessment, three items related to social network, education and financial situation, due to their well-known relationship to health outcomes, have been added, see Additional file 1. For the one-year follow-up visit, an additional three questions capturing the patients trust in treatment and satisfaction with care and treatment and three questions regarding smoking habits, exercising and weight and length have been added, see Additional file 2.

#### Biomolecular analyses

Blood samples will be analyzed using different advanced omics technologies.

#### Clinical serology

Throughout the project, the initial informed consent for the SCAN-B study will grant access to results from the SCAN-B study. This includes traditional routine biochemical analyses performed at the department of clinical chemistry at the different hospitals, at the time of diagnosis and during follow-ups. The subgroup for each tumor will be decided by routine pathological diagnosis and the blood sample analyzed using conventional clinical chemistry tests. Furthermore, we are able to perform additional blood analysis, including measures of inflammation (e.g., IL8, IL6, CRP), as well as stress hormone (cortisol); however, this is dependent on the financial situation.

#### RNA-seq and tumor phenotyping

RNA-seq and tumor phenotyping are performed on tumor samples from the patient in the SCAN-B study and will be analyzed. This includes RNA-seq gene expression measurements and RNA-seq mutation analysis.

### Inclusion criteria

- Newly diagnosed patients with primary breast cancer.
- Patients consented to be included in the SCAN-B study at (Blekinge County Hospital, Central Hospital Växjö, Hallands Hospital Halmstad and Helsingborg Hospital.
- Oral and written consent for the SCAN-B Resilience study.
- Age ≥ 18 years.
- Patients who understand the Swedish language (written and spoken).

### Exclusion criteria

- No diagnosis of breast cancer.
- Not consented to be included in the SCAN-B study.
- Do not understand the Swedish language.

### Data management

All data are registered using an electronic version of the biopsychological assessments and Case Report Form (eCRF) based on the web application Teleform. The data include CD-RISC, SF-36 and data on age, social and socioeconomic variables; at the one-year follow-up, we also collect information regarding smoking, exercise habits, weight and length as well as patients’ trust in the treatment and their satisfaction with the care and treatment. The authority responsible for the database is Lund University.

The blood samples from the patients are stored in Region Skåne Regional biobank, and handling of all personal information is computerized. By giving consent to the study, participants also agree that personal information can be handled according to “Personuppgiftslagen” (PuL). The participants have the right to request information regarding the personal data processing in accordance with the PuL §26.

### Outcomes

#### Primary outcome

- the association between psychological resilience and biomolecular signatures in breast cancer patients.

#### Secondary outcomes

- the association between psychological resilience and quality of life at baseline in breast cancer patients.
- the association between psychological resilience and quality of life one year after diagnosis in breast cancer patients.
- the association between psychological resilience and prognosis in breast cancer patients.
- the association between psychological resilience and clinicopathological characteristics.
- the association between quality of life and clinicopathological characteristics.
- the association between healthcare quality and psychological resilience in breast cancer patients.
- the association between healthcare quality and quality of life one year after diagnosis in breast cancer patients.

### Power calculation

A power analysis has been performed to estimate the required number of patients to include in the study, which enables a statistical power based on the CD-RISC assessment. Briefly, an analytical calculation for a class of simple receiver operating characteristic (ROC) curves has been performed, and the variance of the area under the ROC curve (AUC) was determined to be well described by 4*e*(1-*e*)*V,* where *e* is the AUC expectation value (= effect size) and *V* is the variance for the null distribution*.* To our knowledge, this is a novel approach that allows us to estimate the power for the analysis. This project aims for 80% power, and in this initial analysis, equal prevalence (number of samples) in each of the two classes (high vs. low resilience score) is assumed. The required number of samples as a function of effect size *e* and desired confidence is tabulated below in Table 2. In this study, we will enroll 700–1000 patients, and based on the result below, 40–95 samples are suggested to be set aside as a test set; therefore, this approach leaves an appreciable amount of training samples for machine learning. Based on the number of patients from each hospital, we will have enrolled all patients in 2018. The follow-up visits will therefore be finished in 2019*.*

**Table 2:** The required number of samples as a function of effect size *e* and desired confidence

Effect size	Confidence	No. of required samples for 80% power
0.70	0.95	64
0.70	0.99	95
0.75	0.95	40
0.75	0.99	60

### Statistical methods

The assessment responses (CD-RISC) will first be analyzed using exploratory and confirmatory factor analyses [20], establishing the validity of the factor structure and psychometric properties in a Swedish cohort of women with breast cancer. As reference material, we will include a matched cohort of healthy women who were enrolled in the BIG-3 study [21], an open prospective longitudinal cohort study in the county of Skåne. Preferably, the previously discussed factors will be validated through confirmatory factor analysis in this study material, as described earlier [22], or – if need to be – new factors will be extracted by conventional factor analysis. The relationship to quality of life as well as explanatory and confounding variables will be explored with adequate statistical measures. Based on the confirmatory factor analysis, the factors found to have the best explanatory power will be used in relation to biomolecular markers. Machine learning algorithms for binary classification, e.g., support vector machine, random forest and combat normalization, will be performed by our experienced bioinformaticians, while epigenetic analysis will utilize the supplier’s recommendations performed in R or RStudio. The ROC curves of the predictors will be studied, and as a statistical test, the AUC will be used. In all of the above analyses, confounding factors such as age, stage, clinicopathological factors, including systemic therapy, and the type of other treatment modalities, etc. will be considered.

## Discussion

### Significance

The proposed project suggests a novel way of thinking in that it couples a psychological behavior to biomolecular parameters in a prospective monitoring project. Understanding the molecular mechanisms that are interacting with complex behavior, such as psychological resilience, would help to identify novel treatment strategies and provide knowledge about how to personalize psychosocial support for cancer patients. The importance and need of a study such as this was recently emphasized in 16 independent quantitative studies, including 3250 patients with different cancer diagnoses [23].

From a clinical perspective, it is most important to identify those with the least capacity to manage the stressors of cancer and the subsequent treatment regimens and who are therefore more at risk for a poor outcome and decreased quality of life. The identification of biomolecular signatures, deciphered from the collected blood samples, and their association with high or low psychological resilience could potentially have a major impact for the patient since it could open up a novel avenue of medical as well as psychological intervention options. Here, the rationale is that a biomolecular signature associated with low psychological resilience could eventually be treated and turned into a bodily state of mind that is more associated with high psychological resilience. We cannot treat what we cannot measure, and such interventions could be personalized psychosocial interventions or new medical treatments. For example, whether existing FDA-approved pharmaceutical therapies, such as DNA methyl-transferases inhibitors (5-azacytidine or 5-aza-20-deoxycytidine), could play a role remains to be seen. A vision is that the armament of cancer therapies, including chemotherapy, radiation, surgery, biological pharmaceuticals, etc., in the future could perhaps be complemented with a novel approach to increase the survival of cancer patients by addressing psychological resilience. This could obviously have an impact on patient care and the patient’s quality of life.

### Current status

A total of four hospitals in southern Sweden are participating (Blekinge County Hospital, Central Hospital Växjö, Hallands Hospital Halmstad and Helsingborg Hospital). The first patient was included in February 2016, and the first one-year follow-up was performed in February 2017. In total (February 2018), 420 patients have consented and are participating in the study.

## Additional files

Additional file 1: Question form for social and socioeconomic variables. (DOCX 15 kb)

Additional file 2: Questions capturing the patients trust in treatment and satisfaction with care and treatment and questions regarding smoking habits, exercising and weight. (DOCX 15 kb)

## Abbreviations

AUC: Area Under the ROC curve
CD-RISC: Connor-Davidson Resilience Scale
CRF: Clinical Research Form
INCA: Information Network for CAncer care
ROC: Receiver Operating Characteristic
SCAN-B: Sweden Cancerome Analysis Network – Breast
SF-36: Short Form (36-item) Health Survey
