# Supplementary material for: A multicenter study investigating the molecular fingerprint of psychological resilience in breast cancer patients: study protocol of the SCAN-B resilience study
Source: BMC Cancer. 2018 Aug 6;18:789. doi: 10.1186/s12885-018-4669-y (PMC6091191; doi:10.1186/s12885-018-4669-y)
Supplement: Supplementary file 1 — Question form for social and socioeconomic variables. (DOCX 15 kb) [file 12885_2018_4669_MOESM1_ESM.docx]

# Additional file 11

1. Please mark your highest education level:

Doctoral education (PhD)

Post-secondary education, 2 years or longer

Post-secondary education less than 2 years

Secondary education

Primary school 9 (10) years

Primary school shorter than 9 years

1. Below is question related to how you live
2. Please mark the alternative that suits you best.

Live together with adult/adults only

Live together with adult/adults and children under 18 years old

Live together with children under 18 years old only

Live alone

1. If you have answered that you are living by your own: Do you live in a living apart relationship?

Yes

No

1. Should you / your household within one month be able to pay an unexpected expense of SEK 11,000 without lending or asking for help?

Yes

No
