# Supplementary material for: A multicenter study investigating the molecular fingerprint of psychological resilience in breast cancer patients: study protocol of the SCAN-B resilience study
Source: BMC Cancer. 2018 Aug 6;18:789. doi: 10.1186/s12885-018-4669-y (PMC6091191; doi:10.1186/s12885-018-4669-y)
Supplement: Supplementary file 2 — Questions capturing the patients trust in treatment and satisfaction with care and treatment and questions regarding smoking habits, exercising and weight. (DOCX 15 kb) [file 12885_2018_4669_MOESM2_ESM.docx]

# Additional file 12

1. To what extent do you feel confident with the treatment you receive/ received for breast cancer?
   1. Not at all
   2. To a lesser extent
   3. To some extent
   4. To a large extent
   5. To a very high degree
2. How satisfied are you with the implementation of the treatment you receive / received for the breast cancer?
   1. Not at all
   2. To a lesser extent
   3. To some extent
   4. To a large extent
   5. To a very high degree
3. How satisfied are you with the individual treatment you received during treatment?
   1. Not at all
   2. To a lesser extent
   3. To some extent
   4. To a large extent
   5. To a very high degree
4. How often do you do any type of physical activity (at least 30 minutes)?
   1. Every day
   2. At least 3-4 times a week
   3. At least 1-2 times per week
   4. Less than once a week
5. What do your smoking habits look like?
   1. I smoke every day
   2. I smoke sometime during the month
   3. I have previously smoked every day for longer periods but do not smoke on a regular basis today
   4. I have never smoked regularly
6. In order for us to calculate your BMI, please fill in
   1. Weight ...... .. kg
   2. Length ....... m
